# Impact of Residential Green Space on Sleep Quality and Sufficiency in Children and Adolescents Residing in Australia and Germany

**DOI:** 10.3390/ijerph17134894

**Published:** 2020-07-07

**Authors:** Xiaoqi Feng, Claudia Flexeder, Iana Markevych, Marie Standl, Joachim Heinrich, Tamara Schikowski, Sibylle Koletzko, Gunda Herberth, Carl-Peter Bauer, Andrea von Berg, Dietrich Berdel, Thomas Astell-Burt

**Affiliations:** 1 School of Public Health and Community Medicine, Faculty of Medicine, UNSW, Sydney NSW 2052, Australia; 2 Population Wellbeing and Environment Research Lab (PowerLab), School of Public Health, Faculty of Social Sciences, University of Wollongong, Wollongong NSW 2522, Australia; thomasab@uow.edu.au; 3 National Institute of Environmental Health, Chinese Center for Disease Control and Prevention, Beijing 100050, China; 4 Menzies Centre for Health Policy, University of Sydney, Sydney 2006, Australia; 5 Institute of Epidemiology, Helmholtz Zentrum München-German Research Center for Environmental Health, Ingolstädter Landstraße 1, 85764 Neuherberg, Germany; claudia.flexeder@helmholtz-muenchen.de (C.F.); iana.markevych@uj.edu.pl (I.M.); Marie.standl@helmholtz-muenchen.de (M.S.); 6 Institute and Clinic for Occupational, Social and Environmental Medicine, University Hospital, LMU Munich, Ziemssenstraße 1, 80336 Munich, Germany; Joachim.Heinrich@med.uni-muenchen.de; 7 Institute of Psychology, Jagiellonian University, 30-060 Krakow, Poland; 8 Allergy and Lung Health Unit, Melbourne School of Population and Global Health, The University of Melbourne, Melbourne 3053, Australia; 9 IUF, Leibniz Research Institute for Environmental Medicine, 40225 Düsseldorf, Germany; Tamara.Schikowski@IUF-Duesseldorf.de; 10 Department of Pediatrics, Dr. von Hauner Children’s Hospital, LMU Klinikum, University of Munich, 80337 Munich, Germany; Sibylle.Koletzko@med.uni-muenchen.de; 11 Department of Pediatrics, Gastroenterology and Nutrition, School of Medicine Collegium Medicum University of Warmia and Mazury, 10-719 Olsztyn, Poland; 12 Department of Environmental Immunology/Core Facility Studies, Helmholtz Centre for Environmental Research-UFZ, 04318 Leipzig, Germany; gunda.herberth@ufz.de; 13 Department of Pediatrics, Technical University of Munich, 80804 Munich, Germany; cpbauer@t-online.de; 14 Research Institute, Department of Pediatrics, Marien-Hospital Wesel, 46483 Wesel, Germany; avb.rodehorst@gmx.de (A.v.B.); berdel.vonberg@t-online.de (D.B.); 15 School of Public Health, Peking Union Medical College and The Chinese Academy of Medical Sciences, Beijing 100730, China

**Keywords:** green space, children, epidemiology, noise, sleep

## Abstract

Increasing evidence suggests adults living in greener areas tend to have more favourable sleep-related outcomes, but children and adolescents are under-researched. We hypothesised that children and adolescents living in greener areas would have better quality and more sufficient levels of sleep on average, especially within the context of high traffic noise exposure. These hypotheses were tested using multilevel logistic regressions fitted on samples from the nationally representative Longitudinal Study of Australian Children (10–11 years old, *n* = 3469, and 14–15 years old, *n* = 2814) and the GINIplus and LISA cohorts (10 years old, *n* = 1461, and 15 years old, *n* = 4172) from the Munich, Wesel, and Leipzig areas of Germany. Questionnaire-based binary indicators of sleep sufficiency and sleep quality in each cohort were assessed with respect to objectively measured green space exposures adjusting for age, sex, and maternal education. Models were augmented with proxy measures of traffic noise and two-way interaction terms to test for effect modification. Cross-tabulations illustrated little convincing evidence of association between green space and insufficient sleep or poor sleep quality in either sample, except for insufficient sleep among 10 year old participants in Germany. These null findings were replicated in adjusted models. The proxy for traffic noise was associated with poor quality sleep in 15 year old participants in Germany, but no convincing evidence of modified association with green space was observed.

## 1. Introduction

Accumulating evidence suggests that poor quality and insufficient sleep in adults may increase the risks for depression [1], obesity [2], neurodegenerative diseases [3], inflammation [4], cardiovascular diseases [5], metabolic syndrome [6], type 2 diabetes [7], injuries [8], and premature mortality [9]. While sufficient sleep of good quality is important for health in general, it is perhaps especially critical during childhood and adolescence [10]. Patterns of sleep-related behaviours and disparities in health outcomes manifesting in adults may be influenced by experiences and interventions during these sensitive developmental periods [11]. Meta-analyses concluded that sleep disturbances increase the risk of major depression and suicide attempts in adolescence [12]. These, in turn, are associated with a higher risk of depression and suicide attempts in adulthood [13]. Meta-analyses have also suggested insufficient and poor quality sleep in childhood and adolescence may increase the risk of obesity [14], reduce levels of cognitive development [15], and also increase the risk of health-related outcomes, such as poor academic performance [16].

Interventions across multiple levels beyond the individual (e.g., at the neighbourhood-scale), are needed to improve levels of quality and sufficient sleep at the population-level [17]. Building and improving of green space (e.g., parks and woodlands) is a potential environmental intervention for which evidence of sleep-related benefits is increasing [18]. Green space may influence sleep via multiple interlinking pathways analogous to those recently outlined for health in general [19]. Green space may serve as a potential modifier of other environmental risk factors of poor quality and insufficient sleep, such as traffic noise [20], excess heat [21], and air pollution [22]. Some work suggests that populations with more green space nearby may be buffered from the harms of noise annoyance, heat, and air pollution [23,24,25]. However, only one study has examined this effect modification pathway thus far, finding no evidence of interaction between green space and self-rated traffic noise in Swedish adults aged 18–79 [26]. Another study reported the mean decibels of outdoor sounds were independent of sleep-green space association, but did not explore effect modification [27].

Psychological benefits of nearby green space may help to restore depleted cognitive capacities and relieve periods of chronic stress [28]. In turn, this can support better sleep [29] in persons living in urban areas who are disproportionately subjected to traffic noise, heat islands, and air pollution in comparison to their rural counterparts [30]. These psychological benefits may support other health-related behaviours, modifying sleep-related outcomes. For example, greener areas may support more socially cohesive communities (e.g., [31,32]) and outdoor physical activity [33], both of which may in turn promote sleep-related outcomes [34,35,36].

Better sleep-related outcomes were found to be associated with greater exposures to nearby green space in a recent systematic review of seven cross-sectional studies and five intervention studies [18]. These promising findings have also found support with recent longitudinal evidence [37]. However, despite the imperative of preventing insufficient and poor quality sleep in children and adolescents to promote long-lasting health gains, only one study has tested for potential association with green space in these younger age groups thus far [38]. Singh and Kenney’s study of the U.S.-based parent/guardian-reported National Survey of Children’s Health found higher odds of sleeping problems on multiple days per week and higher odds of serious sleep problems among children <18 years old with no parks or playgrounds near their homes.

To build on this seminal work, more research is needed on green space and sleep focussed on child and adolescent populations with objectively measured exposure data. This work would also ideally involve an accounting of age-related differences in sleep-related outcomes that are known to occur across childhood and adolescence [39].

Accordingly, the objective of this study was to examine associations between nearby green space and multiple measures of sleep in child and adolescent samples living in different countries—Australia and Germany—sites of previous studies linking nearby green space with favourable child and adolescent mental health outcomes (e.g., [40,41]). Two hypotheses were tested: (1) participants with more green space nearby would tend to have better quality and more sufficient levels of sleep; (2) comparatively weaker or null associations between sleep-related outcomes and nearby traffic noise would be observed among participants with more green space nearby, in contrast to counterparts with less green space.

## 2. Data and Methods

### 2.1. Sources of Study Populations

Data were drawn from samples aged 10–11 years old and 14–15 years old from cohort studies in Australia (the Longitudinal Study of Australian Children, "LSAC") [42] and Germany (the GINIplus and LISA cohorts) [43,44]. The full names of the Germany-based cohorts are the *“German Infant Study on the influence of Nutrition Intervention PLUS environmental and genetic influences on allergy development”* (GINIplus), and the *“Influence of life-style factors on the development of the immune system and allergies in East and West Germany”* (LISA). The Australian Government Department of Social Services and the ethical committee from the University of Wollongong (HREC 2019/015) approved the data analysis from the LSAC study. For the GINIplus and LISA studies, the ethical approval was given by the Bavarian Board of Physicians, the University of Leipzig, and the Board of Physicians of North Rhine-Westphalia.

In Australia, data were extracted from the 2010 (*n* = 4169) and 2014 (*n* = 3537) waves of the kindergarten cohort of the LSAC. Participants in the LSAC had been originally recruited in 2004 at ages 4–5 years from postcodes distributed across Australia (79.4% baseline response rate). Sampling drew from the Medicare enrolment database and a spatially clustered design was used to ensure coverage across all states and territories [45]. A single child per family was eligible to participate, with the majority of data collected initially via home-based face-to-face interviews with parents/guardians and some questions responded to by the children in their later years. The Statistical Area 2 was the smallest geographical scale available for each child. It broadly corresponds to Australian suburbs of approximately 10,000 residents on average [46]. Rural-based participants were excluded.

The GINIplus and LISA studies are ongoing population-based birth cohorts. In the GINIplus study, a total of 5991 healthy full-term neonates were recruited between 1995 and 1998 in obstetric clinics in Munich and Wesel. The GINIplus study comprised an intervention (*n* = 2252) and an observation (*n* = 3739) arm. Newborns with at least one atopic parent or sibling were allocated to the intervention group, whereas newborns without a family history of allergy and children whose parents denied participation in the intervention arm were included in the observation arm. In the LISA study, 3097 healthy newborns from Munich, Wesel, Leipzig, and Bad Honnef were recruited between 1997 and 1999. Data from the 10-year (2005 to 2009) and 15-year (2011 to 2014) follow-ups were used for this study. Eligible participants were recruited and were still residing in the cities of Munich, Wesel, and Leipzig and their surrounding areas. The Munich area comprises two administrative regions of Bavaria (Upper Bavaria and Swabia), the Wesel area comprises two administrative regions of North Rhine-Westphalia (Münster and Düsseldorf), and the Leipzig area comprises two administrative regions of Saxony (Nordsachsen and Leipzig).

The Australia-based data were restricted to participants living in major cities across the country with complete outcome data, resulting in final samples of 3469 (10–11 years old) and 2814 (14–15 years old). The Germany-based data included participants with complete outcome living in urban and rural areas, resulting in final samples of 1461 (10 years old, only LISA) and 4172 (15 years old, GINIplus and LISA). The sex ratio in these final samples was approximately 50:50 in the Australia-based sample (girls 10–11 years old = 1698; 14–15 years old = 1370; boys 10–11 years old = 1771; 14–15 years old = 1444) and the Germany-based sample (girls 10 years old = 694; 15 years old = 2042; boys 10 years old = 767; 15 years old = 2130).

### 2.2. Sleep-Related Outcome Variables

Two sleep-related outcomes were measured in the Australia- and Germany-based samples: (1) sleep sufficiency and (2) sleep quality. In the Australia-based sample, sleep sufficiency was child-reported in response to the question *“During the last month, do you think you usually got enough sleep?” (answers: plenty; just enough; not quite enough; not nearly enough)*. Responses to this question were recoded to (0 =) plenty or just enough, and (1 =) not quite enough or not nearly enough. Sleep quality in the Australia-based sample was also child-reported in response to the question *“During the last month, how well do you feel you have slept in general?” (answers: very well; fairly well; fairly badly; very badly)*. These responses were also recoded to (0 =) very well or fairly well, and (1 =) fairly badly or very badly. Insufficient sleep in the Germany-based data was determined using a cut-point of <9 h at age 10 years and <8 h at age 15 years from parental-reports to the question *“How many hours in total during the day and night does the child sleep on average?”* These cut-points were informed by the National Sleep Foundation’s sleep time duration recommendations [47]. Sleep quality in the Germany-based data was also measured using parental report in response to the question *“Does the child have sleep problems?” (answer: yes; no)*. At age 10 years, information on insufficient sleep and sleep quality was only available in the LISA study.

### 2.3. Green Space Exposure Variables

Green space exposure variables were developed on the basis of 1000 metre (m) buffers around points corresponding to, or providing a surrogate for, the place of residence. In Australia, the population-weighted centroid of each Statistical Area 2 (SA2) was used as a proxy for home location, which helped to improve green space exposure estimates within larger areas on the urban fringe containing geographically clustered populations and countryside. The percentage of green space within each 1000 m buffer was calculated from Mesh Block v2011 data classified by the Australian Bureau of Statistics as “parkland”. A 1000 m buffer-based approach was also taken with the Germany-based data, wherein CORINE land cover and Urban Atlas land use data from 2012 were used to determine the percentage green space around the house address of each child participant in the GINIplus and LISA cohorts. The rationale for combining these two datasets was their coverage and resolution—while Urban Atlas has minimum mapping unit (MMU) of 0.25 ha, it covers only metropolitan areas of Europe (i.e., with population ≥ 100,000), whereas CORINE data cover all of Europe but its MMU is 0.25 ha, which reduces its utility for defining green space indicators within urban areas where pocket parks and gardens are prevalent but not sufficiently captured [48]. Thus, in the combined dataset, Urban Atlas covered urban areas/suburban areas while CORINE covered rural areas. To make the German green space definition comparable to the Australian, green space, urban green spaces, sport and leisure facilities, forests, and shrubs were considered in German data. Since non-linearity of the exposure response relationship was observed, tertiles of green space based upon country-specific distributions were assessed (see Table S1).

### 2.4. Confounders and Effect Modifiers

**Figure 1:**
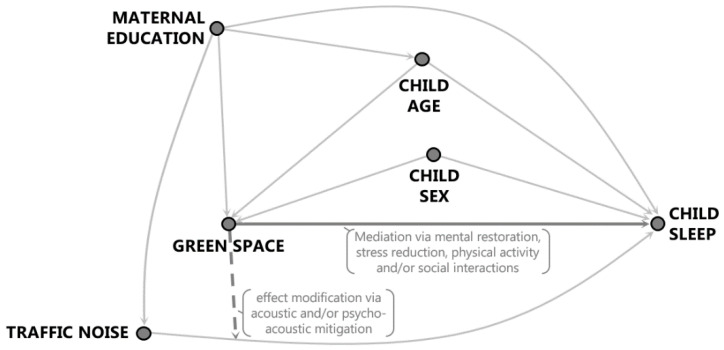
Directed acyclic graph (DAG).

Common causes of sleep-related outcomes in children and adolescents and the probability of living in an area with less green space were determined using published literature to construct a directed acyclic graph (Figure 1). Adjustment for confounding to test the direct effect of green space on child sleep outcomes (Hypothesis 1) took into account child age and sex. Maternal education was also taken into account, not only as a marker of family socioeconomic circumstances that has a significant bearing on where families select to live [49], but also for specific influences on child development that shape sleep trajectories [50], such as impacts of screen-time [51]. These variables were common to data in both countries. Maternal education was measured in both countries according to the number of years the mother spent in education, classified into three categories: (1) less than 10 years, (2) 10 years, and (3) more than 10 years.

Variables to describe or proxy traffic noise, of which green space was proposed to modify association with sleep-related outcomes (Hypothesis 2), were measured differently in both countries. In the Australia-based data, this variable was measured by parental report to the question *“There is heavy traffic on my street or road?” (answers were combined into two categories: (1 = no) “strongly disagree” or “disagree”; (2 = yes) “agree” or “strongly agree”)*. Traffic noise in the Germany-based data was assessed using parental report in response to the question *“On which type of street is your house located?” (answers were combined into two categories: (no) “play street/dead-end street/no road” or “minor road with speed limit (30 km/h)”; (yes) “minor road without speed limit (30 km/h)” or “major road”)*.

### 2.5. Statistical Analysis

Patterns between outcomes and covariates within each sample overall and stratified by sex were assessed using cross-tabulations and chi-squared tests. Hypothesis 1 was tested using multilevel logistic regressions for unadjusted and adjusted associations between each sleep-related outcome and country-specific tertiles of the green space variable within each country. Area of residence (Statistical Area 2) in the Australia-based data was fitted as a random effect to account for spatial clustering of participant reports and green space exposures in the estimation of associations and standard errors [52]. A more complex approach was taken with the Germany-based data due to differences in data available for different age groups. Among the sample aged 10 years, adjustment for study centre (Munich, Wesel, Leipzig) was made in the fixed part of the model. For the sample aged 15 years, a random intercept was fitted for a combination of study (GINIplus, LISA), study centre (Munich, Wesel, Leipzig), and study arm (GINIplus intervention, GINIplus observation).

The fully adjusted multilevel models were extended with the variable of traffic noise and a two-way interaction term fitted with tertiles of green space exposure in order to test Hypothesis 2. A likelihood ratio test was used to determine the degree of change in model fit resulting from including this interaction term.

Sensitivity tests included stratification by sex (both countries) and by study centre (Munich, Wesel, and Leipzig) in the Germany-based data only. The Australian-based data were analysed using Stata v14 (College Station, TX, USA). The Germany-based data were analysed using the statistical software package R, version 3.6.2 (R Core Team: R: A Language and Environment for Statistical Computing. Vienna, Austria: R Foundation for Statistical Computing; 2019 (https://www.R-project.org/)). For the mixed models including random intercepts for Germany-based study/study centre/study arm, we used R-package “lme4” (version 1.1.21) with R-function “glmer”.

## 3. Results

Cross-tabulations illustrated little evidence of association between green space and insufficient sleep or poor quality sleep in either sample, except for insufficient sleep among 10 year old participants in Germany (Table 1). The prevalence of insufficient sleep at age 10 years in the Germany-based sample was lower in green space tertiles 2 and 3 (both 7.61%) compared with tertile 1 (12.47%). These patterns of sleep and green space were the same for girls and boys (Tables S2 and S3).

Differences in the prevalence of sleep-related outcomes by sex between the samples were observed. Levels of insufficient sleep were similar between girls and boys age 10–11 years in both countries. By age 14–15 years, insufficient sleep was markedly higher among girls compared with boys in Australia (24.2% compared with 13.3%) and in Germany (20.0% compared with 14.4%). Poor quality sleep was more common among boys than girls (11.2% compared with 8.2%) aged 10–11 years in Australia, but this pattern had reversed by 14–15 years (6.2% for boys, 13.4% for girls). Levels of poor quality sleep among 10–11 year old participants in Germany were the same for girls and boys (13.8%), but higher among girls than boys aged 14–15 years (15.1% compared with 11.4%). Further sex-related differences are detailed in Tables S2 and S3.

Insufficient sleep was less common among participants of the Germany-based sample with mothers of higher education at age 10–11 years, but not 14–15 years. Insufficient sleep was also higher among participants in the Germany-based sample whose parent reported higher levels of traffic noise. Evidence of sleep-related outcomes being patterned by maternal education and traffic noise in the Australia-based sample was less convincing.

In the Germany-based sample, odds of insufficient sleep at age 10 years were significantly lower among participants in green space tertiles 2 and 3 compared to tertile 1 (Table 2). In other groups, evidence of association between green space and either sleep-related outcome was unconvincing. Odds of poor sleep quality were lower among Australia-based participants aged 10–11 years in tertile 2 compared with tertile 1. No associations were observed between green space and sleep-related outcomes at age 15 years. Adjustments did not change substantially the crude estimates. Sensitivity analyses with additional adjustment for, and then stratification by, categories denoting urban, suburban, and rural areas in the Germany-based cohort yielded comparable results.

Association between sleep-related outcomes and traffic noise was not statistically significant in the Australia-based sample (Table 3). A significantly higher odds of insufficient sleep could be observed among participants exposed to higher levels of traffic noise in the 15 year old Germany-based sample. The fitting of two-way interaction terms provided no convincing evidence for effect modification of sleep-related outcomes and traffic noise across tertiles of green space in either sample.

## 4. Discussion

We hypothesised children and adolescents with more green space nearby would tend to have better quality and more sufficient levels of sleep. Other than for sleep insufficiency among 10 year old participants in Germany, there was little convincing evidence of a direct effect of green space exposure on either sleep outcome in the Australian or German contexts. We also hypothesised potential contingency of association, wherein more favourable sleep outcomes would correlate more strongly with green space within contexts characterised by traffic noise. By contrast, little to no association between sleep and green space would be expected in quieter areas. Our results showed no evidence of traffic noise as an effect modifier of association between sleep and green space in children and adolescents.

Data from recent studies in adults suggested a link between sleep-related outcomes and green space exposure [18]. Only one study [38] extended the focus towards children, reporting affirmative results from parent-reported exposure and outcome data. The current study built upon that emerging evidence with an informal comparison of association between multiple sleep outcomes and objectively measured green space exposure across two countries with varying physical, social, and economic geographies and societal norms. The current study advances the quality of evidence in a number of useful areas. First of all, but for 10 year old children in the Germany-based sample, the generally null findings across multiple outcomes stratified by age and sex in cohorts from two different countries presents reasonably consistent findings of the absence of association between child sleep-related outcomes and green space exposure. It is also notable that the measure of traffic noise was not associated with most of the sleep outcomes, except for sleep quality among 15 year old participants in the Germany-based sample. The latter finding is aligned with work using road noise data [53,54].

It is plausible that some parents may adapt within-household capacities to protect their children from environmental exposures during the night that might disturb their children’s sleep, rendering the need for more green space moot. Such adaptation may also constitute a possible explanation for the largely null association between sleep and traffic noise, although an absence of data meant this hypothesis could not be tested in our study. With the sleep-related outcomes being child-reported and the traffic noise-proxy variable being parent-reported, a lack of association may be a result of parents accommodating their children in quieter rooms in their homes by the process of within-household adaptation. Were parents to sleep in rooms more vulnerable to environmental disturbances as a result, this may contribute to the more consistent findings between sleep-related outcomes and green space exposure observed in studies of adults [18]. Accordingly, the findings from the current study ought not unduly influence the conclusions of studies between green space and adult sleep. The latter are bound up with pressures, stresses, and responsibilities associated with an entirely different stage in the lifecourse (e.g., marital relations, employment, income, housing, child rearing), at which point perhaps the utility of nearby green space may become more salient.

The triangulation of findings from two different countries and analysis of objectively measured green space data linked with two high quality cohort studies constitute some of the main strengths of the current study. Studies of sleep in children and adolescents are recommended to investigate multiple sleep-related outcomes simultaneously [39]. The current study utilised child report (Australia) and parent report (Germany) data to examine distributions of sleep insufficiency and poor sleep quality, revealing potentially consequential age- and sex-related contingencies with respect to covariates such as maternal education. In contrast, similar results across different sleep-related outcome variables in different cohorts also point to triangulation as another strength of this study. Future research involving data from multiple countries may also be important to take stock of documented differences in sleep that manifest across contrasting cultural contexts and how they may modify associations with green space. Differences may not only reflect variations in cultural beliefs and societal norms around the perceived function and meaning of sleep [55,56], but could also render associations between measures of sleep and nearby green space exposure contingent upon geography. For example, work using comparable data found children from nations in southern Europe tend to sleep fewer hours than their counterparts in northern European countries [57]. Therefore, comparisons of results between multiple study countries similar to that which has been conducted in this study would enhance knowledge of association between child sleep and green space exposure through a process of triangulation and documentation of potential heterogeneity as a point of discovery, rather than as a nuisance to be controlled.

In terms of limitations, all analyses were of cross-sectional design, and thus reverse causation cannot be ruled out. Generalisability to other contexts may be limited. This study was restricted to two high income countries (HIC). The results from this study may not be generalisable to middle- and low-income countries (LMIC). Previous work has observed associations between socioeconomic variables and child screen-time differ in magnitude and direction across countries of different socioeconomic circumstances [51]. It may be that such differences also manifest with respect to how different communities experience opportunity to access and preference to live nearby green spaces, in addition to potential differences in beliefs and norms around sleep, resulting in context-specific associations. Accordingly, research on child sleep and green space exposure in other countries is warranted.

Other limitations included single-item measures of sleep sufficiency and quality that were not sensitive to potential differences between weekdays and weekends, and the derived sleep sufficiency variable in the Germany-based study adopted the lower threshold recommended by the National Sleep Foundation (9 h at age 10 years, 8 h at age 15 years). For some unknown number of participants, sleep insufficiency may not be avoided by achieving the minimum threshold. We trialled reclassifying sleep sufficiency in the Germany-based data using an alternative cut-point at 10 h for participants aged 10 years, and 9 h for those aged 15 years, but this increased the prevalence of insufficient sleep considerably to 46.75% and 71.72%, respectively, which seems likely to misclassify many more than the cut-point used in the current analysis. These sleep variables were not identical in each country, limiting comparability of data across contexts. SA2 population-weighted centroids were used as proxies for home addresses and perceived traffic intensity was used as a surrogate marker for traffic-related noise in the Australia-based data. The landcover data in Australia was restricted to discrete urban green spaces such as parks, nature reserves, botanic gardens, and golf courses. Green space exposure derived from this landcover data has been shown to be associated with sleep duration in adults [58]. However, street trees outside of these green spaces were not included in the landcover data. Given the fact that evidence from recent work in adults suggests that tree canopy cover may be particularly important for sleep outcomes [37], this is an important avenue for future research.

The Germany-based data were not available for GINIplus at 10 years, had few participants of low socioeconomic circumstances, and the traffic noise variable was also a proxy via the status and average speed limit of the road nearby where each participant lived. The self-reported noise measurement in the Australian data was similarly limited by self-report. It is no surprise that noise-related annoyances are more common among people chronically exposed to higher levels of actual traffic noise (e.g., [53,59]). Similar variables have also been used as a proxy for air pollution exposure in studies of child respiratory health (e.g., [60,61,62]), although work comparing perceived local traffic intensity with objective measures of air pollution reported weak correlations [63]. It is acknowledged that there may be cultural differences in perceptions of traffic noise that may influence comparability of the data in Germany and Australia, although no consistent data were available to test this hypothesis. Overall, this suggests that there is considerable scope to advance this area of research in terms of more precise measurement of traffic-related noise.

Indirect contrasts in results from two well-recognised cohorts in two countries with broadly comparable outcome, confounders, and objectively measured exposure measures is a strength within the context of a literature replete with single-setting studies. By integrating results from multiple settings and data sources, this affords opportunities for triangulation to strengthen potentially causal inferences. A priori declaration of assumptions in the form of a directed acyclic graph is also important for justifying which variables were adjusted in our regression models. Other strengths included the relatively large sample sizes and balanced sex ratios in both cohorts.

In conclusion, children and adolescents in greener areas did not have higher odds of getting sufficient amounts of, or good quality sleep. These null findings were consistent, regardless of traffic noise exposure. This runs counter to increasing evidence suggesting that green space in cities could support better sleep outcomes in adults [18,37] and one previous study in children [38]. Analyses involving objectively measured child and adolescent sleep outcomes and different types of green space may be important avenues for future research.

## Figures and Tables

**Table 1:** Descriptive statistics for the Australian- and Germany-based samples.

	Australia-Based Sample						Germany-Based Sample					
	10–11 Years Old			14–15 Years Old			10 Years Old			15 Years Old		
	Total	Insufficient Sleep (% yes)	Poor Quality Sleep (% yes)	Total	Insufficient Sleep (% yes)	Poor Quality Sleep (% yes)	Total	Insufficient Sleep (% yes)	Poor Quality Sleep (% yes)	Total	Insufficient Sleep (% yes)	Poor Quality Sleep (% yes)
N	3469	18.56	9.74	2814	18.59	9.74	1461	9.24	13.83	4172	17.16	13.21
Green Space												
Tertile 1	1165	18.03	11.00	921	17.00	8.79	489	12.47	13.50	1392	16.95	12.14
Tertile 2	1165	18.37	8.33	932	20.00	11.05	486	7.61	12.76	1389	17.71	13.97
Tertile 3	1139	19.32	10.01	961	18.21	9.37	486	7.61	15.23	1391	16.82	13.52
*p*-value		0.71	0.10		0.19	0.23		0.01	0.52		0.80	0.33
Sex												
Boys	1771	18.63	11.24	1444	13.30	6.00	767	8.74	13.82	2130	14.41	11.36
Girls	1698	18.49	8.19	1370	24.00	13.43	694	9.80	13.83	2042	20.03	15.13
*p*-value		0.91	<0.01		<0.01	<0.01		0.54	1.00		<0.01	<0.01
Age												
10 years	2384	18.75	9.98									
11 years	1085	18.16	9.22									
*p*-value		0.68	0.48									
14 years				1689	19.24	9.89						
15 years				1125	18.00	9.51						
*p*-value					0.27	0.74						
≤10 years							610	7.21	15.90			
>10 years							851	10.69	12.34			
*p*-value								0.03	0.06			
≤15 years										1901	14.99	12.78
>15 years										2271	18.98	13.56
*p*-value											<0.01	0.49
Maternal Education												
<10 years	186	21.51	14.52	118	13.56	7.63	96	19.79	14.58	431	18.33	13.23
10 years	653	18.53	9.65	502	18.00	10.36	522	11.11	14.75	1635	15.84	13.03
>10 years	2592	18.29	9.49	2132	19.04	9.66	831	6.86	13.12	2087	17.97	13.27
Missing values	38	23.68	5.00	62	19.35	11.00	12	8.33	16.67	19	15.79	21.05
*p*-value		0.55	0.08		0.29	0.66		<0.01	0.68		0.19	0.98
Traffic Noise												
No	2614	18.02	9.41	2097	19.12	10.11	1089	8.45	14.23	3174	16.48	13.42
Yes	821	20.46	10.72	642	16.36	7.94	355	12.11	12.68	958	19.31	12.53
Missing values	34		11.76	75	22.67	14.67	17	0.00	11.76	40	20.00	12.50
*p*-value		0.12	0.27		0.11	0.10		0.05	0.52		0.05	0.51

Note: *p*-values calculated minus categories denoting missing values. Insufficient sleep: In the Australia-based sample, sleep sufficiency was child-reported in response to the question *“During the last month, do you think you usually got enough sleep?” (answers: plenty; just enough; not quite enough; not nearly enough).* Responses to this question were recoded to (0 =) plenty or just enough, and (1 =) not quite enough or not nearly enough. Insufficient sleep in the Germany-based data was determined using a cut-point of <9 h at age 10 years and <8 h at age 15 years from parental reports to the question *“How many hours in total during the day and night does the child sleep on average?”* These cut-points were informed by the National Sleep Foundation’s sleep time duration recommendations (Hirshkowitz et al., 2015). Poor quality sleep: Sleep quality in the Australia-based sample was also child-reported in response to the question *“During the last month, how well do you feel you have slept in general?”* *(answers: very well; fairly well; fairly badly; very badly).* These responses were also recoded to (0 =) very well or fairly well, and (1 =) fairly badly or very badly. Sleep quality in the Germany-based data was also measured using parental report in response to the question *“Does the child have sleep problems?”* *(answer: yes; no)*. At 10 years, information on insufficient sleep and sleep quality was only available in the LISA study. Green space: Australian-based data: tertile 1 = 0–6.6%, tertile 2 = 6.6–14.1%, tertile 3 = 14.1%; Germany-based data tertiles: Munich 10 years: tertile 1: 0–9.7%, tertile 2: 9.7–20.6%, tertile 3: 20.6%, Wesel 10 years: tertile 1: 0–3.9%, tertile 2: 3.9–10.3%, tertile 3: 10.3%, Leipzig 10 years: tertile 1: 0–13.1%, tertile 2: 13.1–24.7%, tertile 3: 24.7%; Munich 15 years: tertile 1: 0–9.2%, tertile 2: 9.2–20.4%, tertile 3: 20.4%, Wesel 15 years: tertile 1: 0–5.5%, tertile 2: 5.5–13.3%, tertile 3: 13.3%, Leipzig 15 years: tertile 1: 0–12.5%, tertile 2: 12.5–24.5%, tertile 3: 24.5%. Traffic noise: In the Australia-based data, traffic noise was measured by parental report to the question *“There is heavy traffic on my street or road?”* *(answers were combined into two categories: (1 = no)* *“strongly* *disagree”* *or* *“disagree”;* *(2 = yes)* *“agree”* *or* *“strongly* *agree”)*. Traffic noise in the Germany-based data was assessed using parental report in response to the question *“On which type of street is your house located?”* *(answers were combined into two categories: (no* *=) “play street/dead-end street/no* *road”* *or* *“minor road with speed limit (30 km/h)”;* *(yes =)* *“minor road without speed limit (30 km/h)”* *or* *“major* *road”)*.

**Table 2:** Associations between sleep-related outcomes and tertiles of green space exposure in the Australia- and Germany-based samples.

	Insufficient Sleep		Poor Quality Sleep	
	Unadjusted	Adjusted	Unadjusted	Adjusted
Australia, 10–11 years				
Green space (ref: Tertile 1)				
Tertile 2	1.025 (0.816, 1.288)	1.029 (0.819, 1.292)	**0.742 (0.562, 0.980)**	**0.749 (0.567, 0.990)**
Tertile 3	1.094 (0.872, 1.373)	1.101 (0.877, 1.382)	0.909 (0.696, 1.187)	0.921 (0.704, 1.204)
Germany, 10 years				
Green space (ref: Tertile 1)				
Tertile 2	**0.578 (0.376, 0.888)**	**0.574 (0.372, 0.885)**	0.937 (0.646, 1.360)	0.952 (0.655, 1.383)
Tertile 3	**0.578 (0.376, 0.888)**	**0.562 (0.363, 0.870)**	1.151 (0.804, 1.648)	1.165 (0.811, 1.673)
Australia, 14–15 years				
Green space (ref: Tertile 1)				
Tertile 2	1.237 (0.975, 1.569)	1.258 (0.987, 1.604)	1.289 (0.948, 1.751)	1.307 (0.959, 1.781)
Tertile 3	1.073 (0.843, 1.365)	1.086 (0.850, 1.388)	1.072 (0.782, 1.468)	1.082 (0.788, 1.486)
Germany, 15 years				
Green space (ref: Tertile 1)				
Tertile 2	1.054 (0.866, 1.283)	1.047 (0.858, 1.277)	1.177 (0.944, 1.469)	1.178 (0.942, 1.472)
Tertile 3	0.989 (0.811, 1.207)	1.000 (0.819, 1.221)	1.135 (0.908, 1.418)	1.153 (0.922, 1.442)

OR = odds ratio; 95% CI = 95% confidence interval. Adjusted models include age, sex, and maternal education. Germany-based samples additionally adjusted for study centre (10 years only) and random intercept for study/study centre/study arm (15 years only). Insufficient sleep: In the Australia-based sample, sleep sufficiency was child-reported in response to the question *“During the last month, do you think you usually got enough sleep?” (answers: plenty; just enough; not quite enough; not nearly enough).* Responses to this question were recoded to (0 =) plenty or just enough, and (1 =) not quite enough or not nearly enough. Insufficient sleep in the Germany-based data was determined using a cut-point of <9 h at age 10 years and <8 h at age 15 years from parental reports to the question *“How many hours in total during the day and night does the child sleep on average?”* These cut-points were informed by the National Sleep Foundation’s sleep time duration recommendations (Hirshkowitz et al., 2015). Poor quality sleep: sleep quality in the Australia-based sample was also child-reported in response to the question *“During the last month, how well do you feel you have slept in general?”* *(answers: very well; fairly well; fairly badly; very badly).* These responses were also recoded to (0 =) very well or fairly well, and (1 =) fairly badly or very badly. Sleep quality in the Germany-based data was also measured using parental report in response to the question *“Does the child have sleep problems?”* *(answer: yes; no)*. At 10 years, information on insufficient sleep and sleep quality was only available in the LISA study. Green space: Australian-based data: tertile 1 = 0–6.6%, tertile 2 = 6.6–14.1%, tertile 3 = 14.1%; Germany-based data tertiles: Munich 10 years: tertile 1: 0–9.7%, tertile 2: 9.7–20.6%, tertile 3: 20.6%, Wesel 10 years: tertile 1: 0–3.9%, tertile 2: 3.9–10.3%, tertile 3: 10.3%, Leipzig 10 years: tertile 1: 0–13.1%, tertile 2: 13.1–24.7%, tertile 3: 24.7%; Munich 15 years: tertile 1: 0–9.2%, tertile 2: 9.2–20.4%, tertile 3: 20.4%, Wesel 15 years: tertile 1: 0–5.5%, tertile 2: 5.5–13.3%, tertile 3: 13.3%, Leipzig 15 years: tertile 1: 0–12.5%, tertile 2: 12.5–24.5%, tertile 3: 24.5 %. Bold = *p* < 0.05.

**Table 3:** Effect modification of associations between sleep-related outcomes and traffic-related noise across strata of green space exposure in the Australia- and Germany-based samples.

	Model	Green Space Tertile 2	Green Space Tertile 3	Traffic Noise (yes)	Green Space Tertile 2 x Traffic Noise (yes)	Green Space Tertile 3 x Traffic Noise (yes)
		Odds Ratio (95% Confidence Interval)				
Insufficient sleep						
Australia, 10–11 years	Independent	1.034 (0.823, 1.298)	1.111 (0.886, 1.394)	1.156 (0.944, 1.415)		
	Interaction	0.942 (0.726, 1.222)	0.969 (0.747, 1.256)	0.851 (0.592, 1.223)	1.441 (0.872, 2.381)	**1.714 (1.041, 2.821)**
Germany, 10 years	Independent	**0.569 (0.368, 0.879)**	**0.552 (0.356, 0.857)**	1.450 (0.978, 2.150)		
	Interaction	0.644 (0.384, 1.082)	0.598 (0.351, 1.017)	1.759 (0.970, 3.191)	0.659 (0.253, 1.717)	0.770 (0.299, 1.983)
Australia, 14–15 years	Independent	1.226 (0.956, 1.573)	1.090 (0.849, 1.399)	0.854 (0.670, 1.087)		
	Interaction	1.168 (0.883, 1.546)	1.047 (0.792, 1.385)	0.740 (0.477, 1.149)	1.249 (0.690, 2.262)	1.207 (0.659, 2.213)
Germany, 15 years	Independent	1.028 (0.842, 1.256)	0.986 (0.807, 1.206)	**1.211 (1.003, 1.462)**		
	Interaction	1.096 (0.869, 1.383)	1.068 (0.846, 1.349)	**1.457 (1.057, 2.009)**	0.780 (0.493, 1.233)	0.732 (0.461, 1.161)
Poor quality sleep						
Australia, 10–11 years	Independent	**0.746 (0.563, 0.988)**	0.931 (0.711, 1.220)	1.111 (0.857, 1.441)		
	Interaction	**0.707 (0.508, 0.983)**	0.929 (0.680, 1.269)	1.047 (0.684, 1.602)	1.220 (0.648, 2.296)	1.004 (0.540, 1.867)
Germany, 10 years	Independent	0.932 (0.639, 1.359)	1.179 (0.820, 1.694)	0.901 (0.628, 1.292)		
	Interaction	0.944 (0.617, 1.445)	1.157 (0.765, 1.750)	0.890 (0.463, 1.711)	0.944 (0.374, 2.384)	1.078 (0.453, 2.567)
Australia, 14–15 years	Independent	1.241 (0.905, 1.703)	1.059 (0.768, 1.460)	0.781 (0.565, 1.080)		
	Interaction	1.146 (0.805, 1.630)	0.992 (0.695, 1.416)	0.597 (0.322, 1.110)	1.505 (0.673, 3.366)	1.414 (0.614, 3.255)
Germany, 15 years	Independent	1.164 (0.931, 1.456)	1.132 (0.904, 1.417)	0.915 (0.735, 1.140)		
	Interaction	**1.293 (1.006, 1.661)**	1.079 (0.833, 1.398)	1.009 (0.685, 1.487)	0.595 (0.339, 1.046)	1.205 (0.713, 2.035)

Models were adjusted for age, sex, and maternal education. Germany-based samples additionally adjusted for study centre (10 years only) and random intercept for study/study centre/study arm (15 years only). Independent = models adjusting for traffic noise but without a two-way interaction term fitted between traffic noise and green space. Interaction = models including a two-way interaction term fitted between traffic noise and green space. Insufficient sleep: in the Australia-based sample, sleep sufficiency was child-reported in response to the question *“During the last month, do you think you usually got enough sleep?” (answers: plenty; just enough; not quite enough; not nearly enough).* Responses to this question were recoded to (0 =) plenty or just enough, and (1 =) not quite enough or not nearly enough. Insufficient sleep in the Germany-based data was determined using a cut-point of <9 h at age 10 years and <8 h at age 15 years from parental reports to the question *“How many hours in total during the day and night does the child sleep on average?”* These cut-points were informed by the National Sleep Foundation’s sleep time duration recommendations (Hirshkowitz et al., 2015). Poor quality sleep: sleep quality in the Australia-based sample was also child-reported in response to the question *“During the last month, how well do you feel you have slept in general?”* *(answers: very well; fairly well; fairly badly; very badly)*. These responses were also recoded to (0 =) very well or fairly well, and (1 =) fairly badly or very badly. Sleep quality in the Germany-based data was also measured using parental report in response to the question *“Does the child have sleep problems?”* *(answer: yes; no)*. At 10 years, information on insufficient sleep and sleep quality was only available in the LISA study. Green space: Australian-based data: tertile 1 = 0–6.6%, tertile 2 = 6.6–14.1%, tertile 3 = 14.1%; Germany-based data tertiles: Munich 10 years: tertile 1: 0–9.7%, tertile 2: 9.7–20.6%, tertile 3: 20.6%, Wesel 10 years: tertile 1: 0–3.9%, tertile 2: 3.9–10.3%, tertile 3: 10.3%, Leipzig 10 years: tertile 1: 0–13.1%, tertile 2: 13.1–24.7%, tertile 3: 24.7%; Munich 15 years: tertile 1: 0–9.2%, tertile 2: 9.2–20.4%, tertile 3: 20.4%, Wesel 15 years: tertile 1: 0–5.5%, tertile 2: 5.5–13.3%, tertile 3: 13.3%, Leipzig 15 years: tertile 1: 0–12.5%, tertile 2: 12.5–24.5%, tertile 3: 24.5%. Bold = *p* < 0.05.

## Supplementary Materials

The following are available online at https://www.mdpi.com/1660-4601/17/13/4894/s1, Table S1: Green space tertile cut-points; Table S2: Descriptive Statistics for the Australian- and Germany-based samples, girls only; Table S3: Descriptive Statistics for the Australian- and Germany-based samples, boys only.
