# Supplementary material for: Impact of Residential Green Space on Sleep Quality and Sufficiency in Children and Adolescents Residing in Australia and Germany
Source: Int J Environ Res Public Health. 2020 Jul 7;17(13):4894. doi: 10.3390/ijerph17134894 (PMC7369701; doi:10.3390/ijerph17134894)
Supplement: Supplementary file 1 [file ijerph-17-04894-s001.pdf]

Table S1: Green space tertile cut-points.

Australian-based green space data	Tertile cut-points
Tertile 1	0–6.6%
Tertile 2	= > 14.1%
Tertile 3	= > 14.1%
**Germany-based green space data**	**Tertile cut-points**
Munich 10y:	
Tertile 1	0–9.7%
Tertile 2	9.7–20.6%
Tertile 3	= > 20.6%
Munich 15y	
Tertile 1	0–9.2%
Tertile 2	9.2–20.4%
Tertile 3	= > 20.4%
Wesel 10y	
Tertile 1	0–3.9%
Tertile 2	3.9–10.3%
Tertile 3	= > 10.3%
Munich 15y	
Tertile 1	0–5.5%
Tertile 2	5.5–13.3%
Tertile 3	= > 13.3%
Leipzig 10y	
Tertile 1	0–13.1%
Tertile 2	13.1–24.7%
Tertile 3	=>24.7%
Leipzig 15y	
Tertile 1	0–12.5%
Tertile 2	12.5–24.5%
Tertile 3	=>24.5%

Table S2: Descriptive Statistics for the Australian- and Germany-based samples, girls only.

	Australia-based sample						Germany-based sample					
	10–11y			14–15y			10–11y			14–15y		
	Total	Insufficient Sleep (% yes)	Poor Quality Sleep (% yes)	Total	Insufficient Sleep (% yes)	Poor Quality Sleep (% yes)	Total	Insufficient Sleep (% yes)	Poor Quality Sleep (% yes)	Total	Insufficient Sleep (% yes)	Poor Quality Sleep (% yes)
n	1,698	18.49	8.19	1,370	24.16	13.43	694	9.80	13.83	2,042	20.03	15.13
Green Space												
Tertile 1	566	16.43	8.30	458	21.18	12.01	238	13.03	13.45	678	19.03	14.01
Tertile 2	573	19.20	7.16	448	27.23	15.18	236	8.05	13.98	683	20.94	17.28
Tertile 3	559	19.86	9.12	464	24.14	13.15	220	8.18	14.09	681	20.12	14.10
p-value		0.29	0.48		0.10	0.37		0.12	0.98		0.68	0.16
Age												
10y/14y	1,170	18.38	8.21	824	24.27	12.74	289	6.57	14.88	917	17.88	14.61
11y/15y	528	18.75	8.14	546	23.99	14.47	405	12.10	13.09	1,125	21.78	15.56
p-value		0.85	0.97		0.91	0.36		0.02	0.57		0.03	0.60
Maternal Education												
<10 years	99	23.23	12.12	60	16.67	6.67	48	22.92	16.67	207	19.81	14.01
10 years	337	19.29	7.72	245	25.31	15.51	250	12.40	12.80	799	17.90	14.27
>10 years	1,246	18.06	8.11	1,043	24.16	13.14	390	6.67	14.10	1,027	21.81	15.97
Missing values	16	6.25	0.00	22	31.82	22.73	6	0.00	16.67	9	11.11	22.22
p-value		0.42	0.35		0.37	0.19		<0.01	0.75		0.12	0.54
Traffic Noise												
No	1,302	18.43	7.53	1,036	25.00	14.48	509	8.64	14.73	1,543	19.70	15.29
Yes	379	19.00	10.29	299	20.07	8.70	175	13.71	11.43	482	21.16	14.52
Missing values	17	11.76	11.76	35	34.29	22.86	10	0.00	10.00	17	17.65	17.65
p-value		0.80	0.08		0.08	<0.01		0.07	0.33		0.53	0.73

Note: *p*-values calculated minus categories denoting missing values.

Insufficient sleep: In the Australia-based sample, sleep sufficiency was child-reported in response to the question *"During the last month, do you think you usually got enough sleep? [answers: plenty; just enough; not quite enough; not nearly enough].* Responses to this question were recoded to (0=) plenty or just enough, and (1=) not quite enough or not nearly enough. Insufficient sleep in the Germany-based data was determined using a cut-point of <9 hours at age 10y and <8 hours at age 15y from parental-reports to the question *"How many hours does the child sleep?"* These cut-points were informed by the National Sleep Foundation's sleep time duration recommendations (Hirshkowitz et al., 2015).
Poor quality sleep: Sleep quality in the Australia-based sample was also child-reported in response to the question *"During the last month, how well do you feel you have slept in general?" [answers: very well; fairly well; fairly badly; very badly].* These responses were also recoded to (0=) very well or fairly well, and (1=) fairly badly or very badly. Sleep quality in the Germany-based data was also measured using parental report in response to the question *"Does the child have sleep problems?" [answer: yes; no]*. At 10 years, information on insufficient sleep and sleep quality was only available in the LISA study.
Green space: Australian-based data tertile 1=0–6.6%, tertile 2=6.6%-14.1%, tertile 3=14.1%; Germany-based data tertiles: Munich 10y: tertile 1: 0–9.7%, tertile 2: 9.7–20.6%, tertile 3: 20.6%, Wesel 10y: tertile 1: 0–3.9%, tertile 2: 3.9–10.3%, tertile 3: 10.3%, Leipzig 10y: tertile 1: 0–13.1%, tertile 2: 13.1–24.7%, tertile 3: 24.7%; Munich 15y: tertile 1: 0–9.2%, tertile 2: 9.2–20.4%, tertile 3: 20.4%, Wesel 15y: tertile 1: 0–5.5%, tertile 2: 5.5–13.3%, tertile 3: 13.3%, Leipzig 15y: tertile 1: 0–12.5%, tertile 2: 12.5–24.5%, tertile 3: 24.5%).
Traffic noise: In the Australia-based data traffic noise was measured by parental report to the question "There is heavy traffic on my street or road?" [answers were combined into two categories: (1=no) 'strongly disagree' or 'disagree'; (2=yes) 'agree' or 'strongly agree']. Traffic noise in the Germany-based data was assessed using parental report in response to the question "On which type of street is your house located?" [answers were combined into two categories: (no=) 'play street/dead-end street/no road' or 'minor road with speed limit (30km/h)'; (yes=) 'minor road without speed limit (30km/h)' or major road'].

Table S3: Descriptive Statistics for the Australian- and Germany-based samples, boys only.

	Australia-based sample						Germany-based sample					
	10–11y			14–15y			10–11y			14–15y		
	Total	Insufficient Sleep (% yes)	Poor Quality Sleep (% yes)	Total	Insufficient Sleep (% yes)	Poor Quality Sleep (% yes)	Total	Insufficient Sleep (% yes)	Poor Quality Sleep (% yes)	Total	Insufficient Sleep (% yes)	Poor Quality Sleep (% yes)
n	1,771	18.63	11.24	1,444	13.30	6.23	767	8.74	13.82	2,130	14.41	11.36
Green Space												
Tertile 1	599	19.53	13.00	463	13.17	5.62	251	11.95	13.55	714	14.99	10.36
Tertile 2	592	17.57	9.46	484	14.05	7.23	250	7.20	11.60	706	14.59	10.76
Tertile 3	580	18.79	10.86	497	13.00	5.84	266	7.14	16.17	710	13.66	12.96
p-value		0.68	0.10		0.82	0.53		0.09	0.32		0.77	0.25
Age												
10y/14y	1,214	19.11	11.70	865	14.45	7.00	321	7.79	16.82	984	12.30	11.08
11y/15y	557	17.59	10.23	579	12.00	4.84	446	9.42	11.66	1,146	16.23	11.61
p-value		0.58	0.37		0.11	0.07		0.51	0.05		0.01	0.75
Maternal Education												
<10 years	87	19.54	17.24	58	10.34	8.62	48	16.67	12.50	224	16.96	12.50
10 years	316	17.72	11.71	257	10.51	5.45	272	9.93	16.54	836	13.88	11.84
>10 years	1,346	18.50	10.77	1,089	14.14	6.34	441	7.03	12.24	1,060	14.25	10.66
Missing values	22	36.36	9.09	40	12.50	5.00	6	16.67	16.67	10	20.00	20.00
p-value		0.91	0.17		0.24	0.65		0.05	0.26		0.50	0.61
Traffic Noise												
No	1,312	17.61	11.28	1061	13.38	5.84	580	8.28	13.79	1,631	13.43	11.65
Yes	442	21.72	11.00	343	13.12	7.29	180	10.56	13.89	476	17.44	10.50
Missing values	17	17.65	11.76	40	12.50	7.50	7	0.00	14.29	23	21.74	8.70
p-value		0.06	0.91		0.90	0.60		0.43	1.00		0.03	0.54

Note: *p*-values calculated minus categories denoting missing values.

Insufficient sleep: In the Australia-based sample, sleep sufficiency was child-reported in response to the question *"During the last month, do you think you usually got enough sleep? [answers: plenty; just enough; not quite enough; not nearly enough].* Responses to this question were recoded to (0=) plenty or just enough, and (1=) not quite enough or not nearly enough. Insufficient sleep in the Germany-based data was determined using a cut-point of <9 hours at age 10y and <8 hours at age 15y from parental-reports to the question *"How many hours does the child sleep?"* These cut-points were informed by the National Sleep Foundation's sleep time duration recommendations (Hirshkowitz et al., 2015).

Poor quality sleep: Sleep quality in the Australia-based sample was also child-reported in response to the question *"During the last month, how well do you feel you have slept in general?" [answers: very well; fairly well; fairly badly; very badly].* These responses were also recoded to (0=) very well or fairly well, and (1=) fairly badly or very badly. Sleep quality in the Germany-based data was also measured using parental report in response to the question *"Does the child have sleep problems?" [answer: yes; no].* At 10 years, information on insufficient sleep and sleep quality was only available in the LISA study.

Green space: Australian-based data tertile 1=0–6.6%, tertile 2=6.6%-14.1%, tertile 3=14.1%; Germany-based data tertiles: Munich 10y: tertile 1: 0–9.7%, tertile 2: 9.7–20.6%, tertile 3: 20.6%, Wesel 10y: tertile 1: 0–3.9%, tertile 2: 3.9–10.3%, tertile 3: 10.3%, Leipzig 10y: tertile 1: 0–13.1%, tertile 2: 13.1–24.7%, tertile 3: 24.7%; Munich 15y: tertile 1: 0–9.2%, tertile 2: 9.2–20.4%, tertile 3: 20.4%, Wesel 15y: tertile 1: 0–5.5%, tertile 2: 5.5–13.3%, tertile 3: 13.3%, Leipzig 15y: tertile 1: 0–12.5%, tertile 2: 12.5–24.5%, tertile 3: 24.5%).

Traffic noise: In the Australia-based data was measured by parental report to the question "There is heavy traffic on my street or road?" [answers were combined into two categories: (1=no) 'strongly disagree' or 'disagree'; (2=yes) 'agree' or 'strongly agree']. Traffic noise in the Germany-based data was assessed using parental report in response to the question "On which type of street is your house located?" [answers were combined into two categories: (no=) 'play street/dead-end street/no road' or 'minor road with speed limit (30km/h)'; (yes=) 'minor road without speed limit (30km/h)' or 'major road'].
